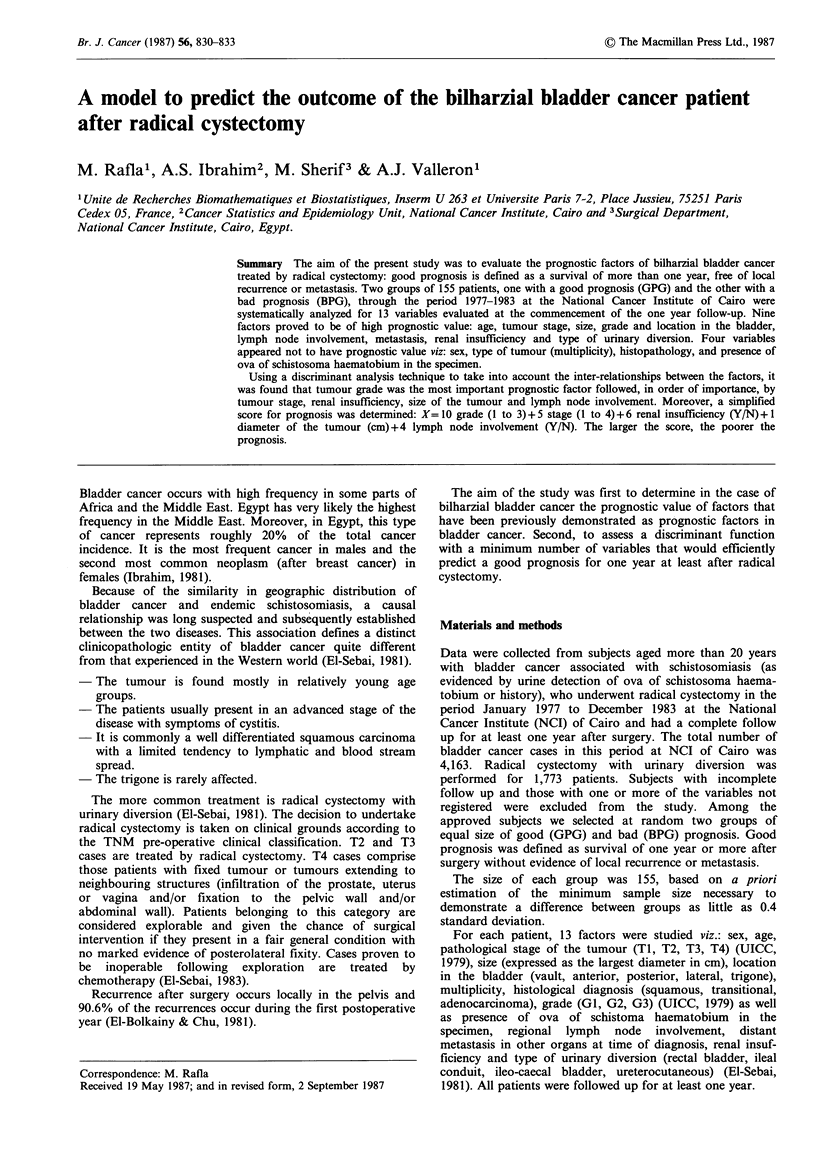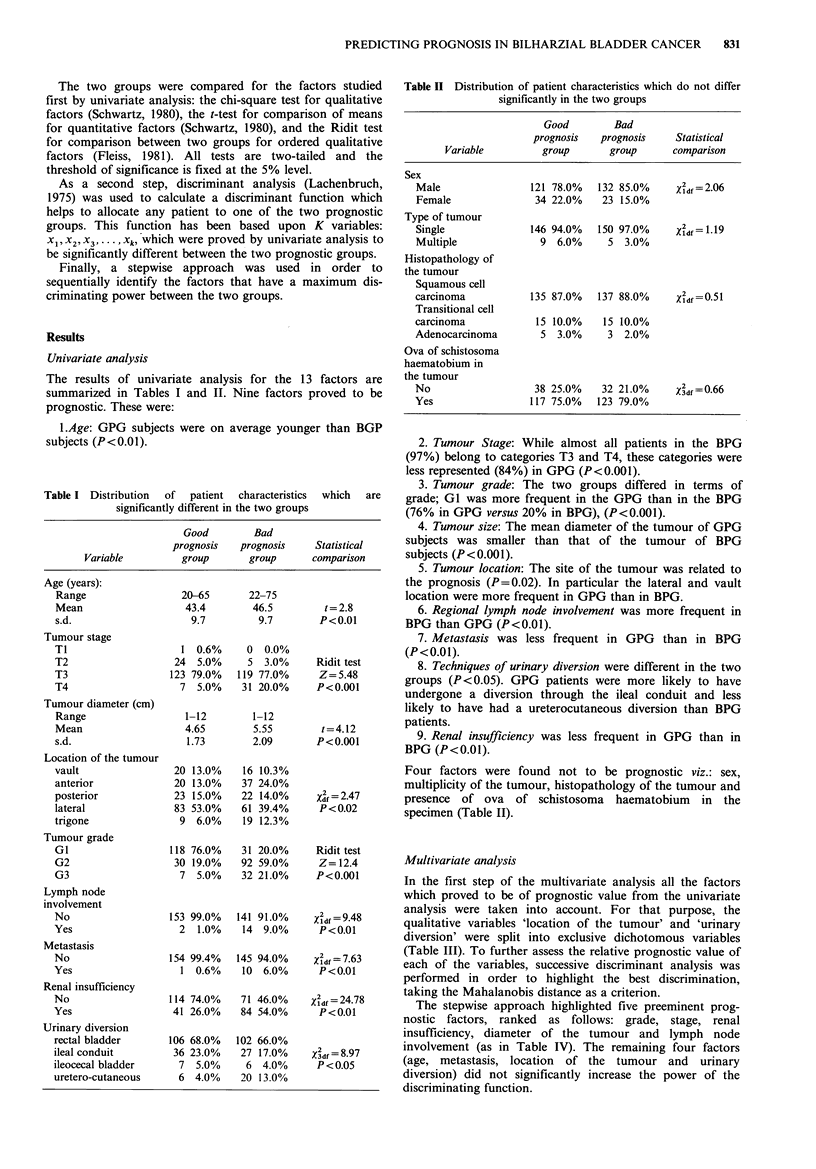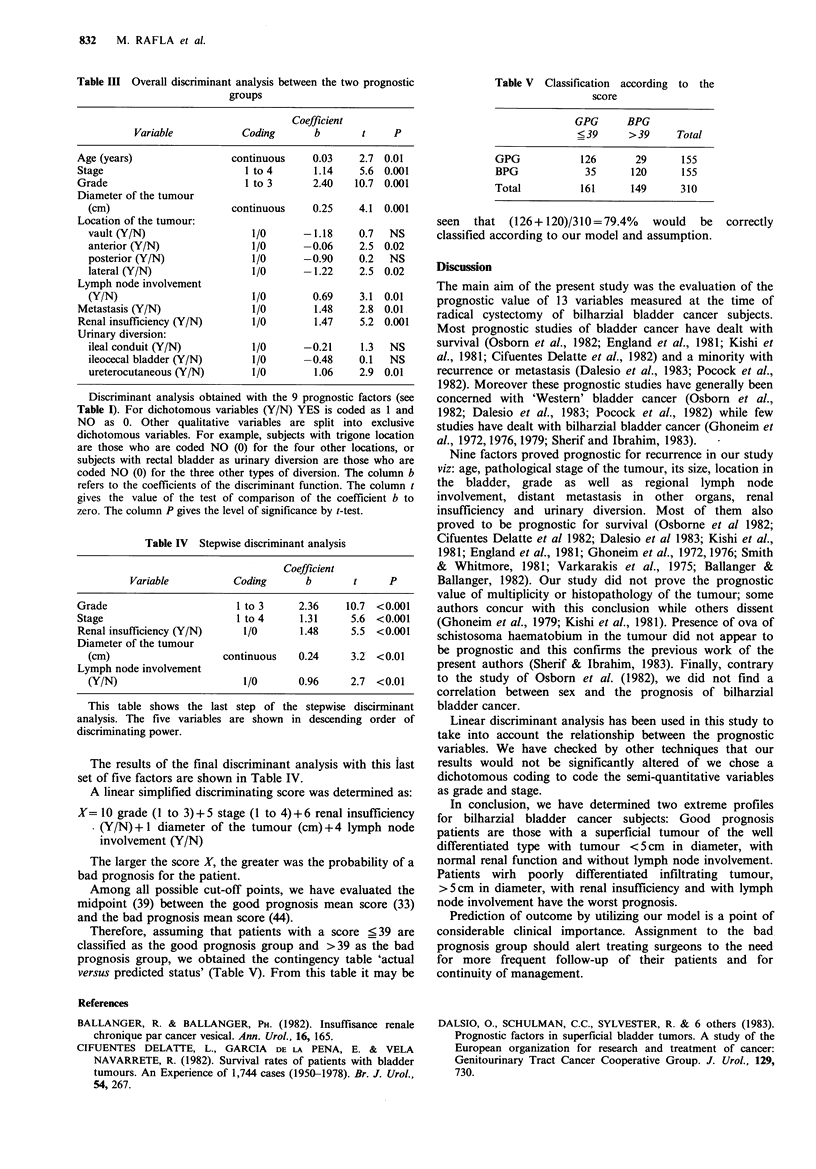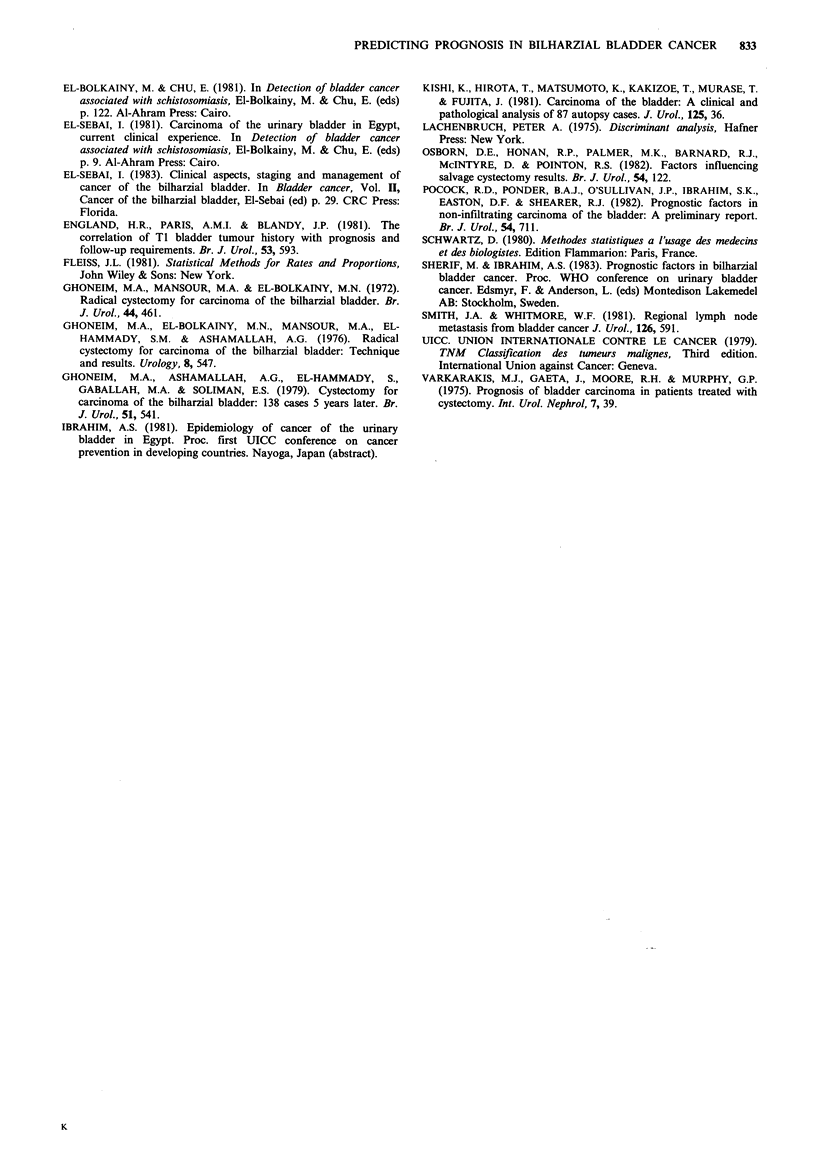# A model to predict the outcome of the bilharzial bladder cancer patient after radical cystectomy.

**DOI:** 10.1038/bjc.1987.299

**Published:** 1987-12

**Authors:** M. Rafla, A. S. Ibrahim, M. Sherif, A. J. Valleron

**Affiliations:** Unite de Recherches Biomathematiques et Biostatistiques, Inserm U 263 et Universite Paris, France.

## Abstract

The aim of the present study was to evaluate the prognostic factors of bilharzial bladder cancer treated by radical cystectomy: good prognosis is defined as a survival of more than one year, free of local recurrence or metastasis. Two groups of 155 patients, one with a good prognosis (GPG) and the other with a bad prognosis (BPG), through the period 1977-1983 at the National Cancer Institute of Cairo were systematically analyzed for 13 variables evaluated at the commencement of the one year follow-up. Nine factors proved to be of high prognostic value: age, tumour stage, size, grade and location in the bladder, lymph node involvement, metastasis, renal insufficiency and type of urinary diversion. Four variables appeared not to have prognostic value viz: sex, type of tumour (multiplicity), histopathology, and presence of ova of schistosoma haematobium in the specimen. Using a discriminant analysis technique to take into account the inter-relationships between the factors, it was found that tumour grade was the most important prognostic factor followed, in order of importance, by tumour stage, renal insufficiency, size of the tumour and lymph node involvement. Moreover, a simplified score for prognosis was determined: X = 10 grade (1 to 3) +5 stage (1 to 4) +6 renal insufficiency (Y/N) +1 diameter of the tumour (cm) +4 lymph node involvement (Y/N). The larger the score, the poorer the prognosis.


 © The Macmillan Press Ltd., 1987

### A model to predict the outcome of the bilharzial bladder cancer patient after radical cystectomy

M. Rafla[1], A.S. Ibrahim[2], M. Sherif[3] & A.J. Valleron[1]

[1]*Unite de Recherches Biomathematiques et Biostatistiques, Inserm U 263 et Universite Paris 7-2, Place Jussieu, 75251 Paris Cedex 05, France,* [2]*Cancer Statistics and Epidemiology Unit, National Cancer Institute, Cairo and* [3]*Surgical Department, National Cancer Institute, Cairo, Egypt.*

**Summary** The aim of the present study was to evaluate the prognostic factors of bilharzial bladder cancer treated by radical cystectomy: good prognosis is defined as a survival of more than one year, free of local recurrence or metastasis. Two groups of 155 patients, one with a good prognosis (GPG) and the other with a bad prognosis (BPG), through the period 1977–1983 at the National Cancer Institute of Cairo were systematically analyzed for 13 variables evaluated at the commencement of the one year follow-up. Nine factors proved to be of high prognostic value: age, tumour stage, size, grade and location in the bladder, lymph node involvement, metastasis, renal insufficiency and type of urinary diversion. Four variables appeared not to have prognostic value *viz*: sex, type of tumour (multiplicity), histopathology, and presence of ova of schistosoma haematobium in the specimen.

Using a discriminant analysis technique to take into account the inter-relationships between the factors, it was found that tumour grade was the most important prognostic factor followed, in order of importance, by tumour stage, renal insufficiency, size of the tumour and lymph node involvement. Moreover, a simplified score for prognosis was determined: $X=10$ grade (1 to 3)+5 stage (1 to 4)+6 renal insufficiency (Y/N)+1 diameter of the tumour (cm)+4 lymph node involvement (Y/N). The larger the score, the poorer the prognosis.

Bladder cancer occurs with high frequency in some parts of Africa and the Middle East. Egypt has very likely the highest frequency in the Middle East. Moreover, in Egypt, this type of cancer represents roughly 20% of the total cancer incidence. It is the most frequent cancer in males and the second most common neoplasm (after breast cancer) in females (Ibrahim, 1981).

Because of the similarity in geographic distribution of bladder cancer and endemic schistosomiasis, a causal relationship was long suspected and subsequently established between the two diseases. This association defines a distinct clinicopathologic entity of bladder cancer quite different from that experienced in the Western world (El-Sebai, 1981).

- — The tumour is found mostly in relatively young age groups.
- — The patients usually present in an advanced stage of the disease with symptoms of cystitis.
- — It is commonly a well differentiated squamous carcinoma with a limited tendency to lymphatic and blood stream spread.
- — The trigone is rarely affected.

The more common treatment is radical cystectomy with urinary diversion (El-Sebai, 1981). The decision to undertake radical cystectomy is taken on clinical grounds according to the TNM pre-operative clinical classification. T2 and T3 cases are treated by radical cystectomy. T4 cases comprise those patients with fixed tumour or tumours extending to neighbouring structures (infiltration of the prostate, uterus or vagina and/or fixation to the pelvic wall and/or abdominal wall). Patients belonging to this category are considered explorable and given the chance of surgical intervention if they present in a fair general condition with no marked evidence of posterolateral fixity. Cases proven to be inoperable following exploration are treated by chemotherapy (El-Sebai, 1983).

Recurrence after surgery occurs locally in the pelvis and 90.6% of the recurrences occur during the first postoperative year (El-Bolkainy & Chu, 1981).

Correspondence: M. Rafla
Received 19 May 1987; and in revised form, 2 September 1987

The aim of the study was first to determine in the case of bilharzial bladder cancer the prognostic value of factors that have been previously demonstrated as prognostic factors in bladder cancer. Second, to assess a discriminant function with a minimum number of variables that would efficiently predict a good prognosis for one year at least after radical cystectomy.

### Materials and methods

Data were collected from subjects aged more than 20 years with bladder cancer associated with schistosomiasis (as evidenced by urine detection of ova of schistosoma haematobium or history), who underwent radical cystectomy in the period January 1977 to December 1983 at the National Cancer Institute (NCI) of Cairo and had a complete follow up for at least one year after surgery. The total number of bladder cancer cases in this period at NCI of Cairo was 4,163. Radical cystectomy with urinary diversion was performed for 1,773 patients. Subjects with incomplete follow up and those with one or more of the variables not registered were excluded from the study. Among the approved subjects we selected at random two groups of equal size of good (GPG) and bad (BPG) prognosis. Good prognosis was defined as survival of one year or more after surgery without evidence of local recurrence or metastasis.

The size of each group was 155, based on *a priori* estimation of the minimum sample size necessary to demonstrate a difference between groups as little as 0.4 standard deviation.

For each patient, 13 factors were studied *viz.*: sex, age, pathological stage of the tumour (T1, T2, T3, T4) (UICC, 1979), size (expressed as the largest diameter in cm), location in the bladder (vault, anterior, posterior, lateral, trigone), multiplicity, histological diagnosis (squamous, transitional, adenocarcinoma), grade (G1, G2, G3) (UICC, 1979) as well as presence of ova of schistosoma haematobium in the specimen, regional lymph node involvement, distant metastasis in other organs at time of diagnosis, renal insufficiency and type of urinary diversion (rectal bladder, ileal conduit, ileo-caecal bladder, ureterocutaneous) (El-Sebai, 1981). All patients were followed up for at least one year.

The two groups were compared for the factors studied first by univariate analysis: the chi-square test for qualitative factors (Schwartz, 1980), the $t$-test for comparison of means for quantitative factors (Schwartz, 1980), and the Ridit test for comparison between two groups for ordered qualitative factors (Fleiss, 1981). All tests are two-tailed and the threshold of significance is fixed at the 5% level.

As a second step, discriminant analysis (Lachenbruch, 1975) was used to calculate a discriminant function which helps to allocate any patient to one of the two prognostic groups. This function has been based upon $K$ variables: $x_1, x_2, x_3, \ldots, x_k$, which were proved by univariate analysis to be significantly different between the two prognostic groups.

Finally, a stepwise approach was used in order to sequentially identify the factors that have a maximum discriminating power between the two groups.

### Results

### *Univariate analysis*

The results of univariate analysis for the 13 factors are summarized in Tables I and II. Nine factors proved to be prognostic. These were:

1. *Age*: GPG subjects were on average younger than BGP subjects ($P<0.01$).

**Table I** Distribution of patient characteristics which are significantly different in the two groups

| *Variable* | *Good prognosis group* | *Bad prognosis group* | *Statistical comparison* |
|---|---|---|---|
| Age (years): | | | |
| Range | 20–65 | 22–75 | |
| Mean | 43.4 | 46.5 | $t=2.8$ |
| s.d. | 9.7 | 9.7 | $P<0.01$ |
| Tumour stage | | | |
| T1 | 1 0.6% | 0 0.0% | |
| T2 | 24 5.0% | 5 3.0% | Ridit test |
| T3 | 123 79.0% | 119 77.0% | $Z=5.48$ |
| T4 | 7 5.0% | 31 20.0% | $P<0.001$ |
| Tumour diameter (cm) | | | |
| Range | 1–12 | 1–12 | |
| Mean | 4.65 | 5.55 | $t=4.12$ |
| s.d. | 1.73 | 2.09 | $P<0.001$ |
| Location of the tumour | | | |
| vault | 20 13.0% | 16 10.3% | |
| anterior | 20 13.0% | 37 24.0% | |
| posterior | 23 15.0% | 22 14.0% | $\chi^2_{df}=2.47$ |
| lateral | 83 53.0% | 61 39.4% | $P<0.02$ |
| trigone | 9 6.0% | 19 12.3% | |
| Tumour grade | | | |
| G1 | 118 76.0% | 31 20.0% | Ridit test |
| G2 | 30 19.0% | 92 59.0% | $Z=12.4$ |
| G3 | 7 5.0% | 32 21.0% | $P<0.001$ |
| Lymph node involvement | | | |
| No | 153 99.0% | 141 91.0% | $\chi^2_{1df}=9.48$ |
| Yes | 2 1.0% | 14 9.0% | $P<0.01$ |
| Metastasis | | | |
| No | 154 99.4% | 145 94.0% | $\chi^2_{1df}=7.63$ |
| Yes | 1 0.6% | 10 6.0% | $P<0.01$ |
| Renal insufficiency | | | |
| No | 114 74.0% | 71 46.0% | $\chi^2_{1df}=24.78$ |
| Yes | 41 26.0% | 84 54.0% | $P<0.01$ |
| Urinary diversion | | | |
| rectal bladder | 106 68.0% | 102 66.0% | |
| ileal conduit | 36 23.0% | 27 17.0% | $\chi^2_{3df}=8.97$ |
| ileocecal bladder | 7 5.0% | 6 4.0% | $P<0.05$ |
| uretero-cutaneous | 6 4.0% | 20 13.0% | |

**Table II** Distribution of patient characteristics which do not differ significantly in the two groups

| *Variable* | *Good prognosis group* | *Bad prognosis group* | *Statistical comparison* |
|---|---|---|---|
| Sex | | | |
| Male | 121 78.0% | 132 85.0% | $\chi^2_{1df}=2.06$ |
| Female | 34 22.0% | 23 15.0% | |
| Type of tumour | | | |
| Single | 146 94.0% | 150 97.0% | $\chi^2_{1df}=1.19$ |
| Multiple | 9 6.0% | 5 3.0% | |
| Histopathology of the tumour | | | |
| Squamous cell carcinoma | 135 87.0% | 137 88.0% | $\chi^2_{1df}=0.51$ |
| Transitional cell carcinoma | 15 10.0% | 15 10.0% | |
| Adenocarcinoma | 5 3.0% | 3 2.0% | |
| Ova of schistosoma haematobium in the tumour | | | |
| No | 38 25.0% | 32 21.0% | $\chi^2_{3df}=0.66$ |
| Yes | 117 75.0% | 123 79.0% | |

2. *Tumour Stage*: While almost all patients in the BPG (97%) belong to categories T3 and T4, these categories were less represented (84%) in GPG ($P<0.001$).

3. *Tumour grade*: The two groups differed in terms of grade; G1 was more frequent in the GPG than in the BPG (76% in GPG *versus* 20% in BPG), ($P<0.001$).

4. *Tumour size*: The mean diameter of the tumour of GPG subjects was smaller than that of the tumour of BPG subjects ($P<0.001$).

5. *Tumour location*: The site of the tumour was related to the prognosis ($P=0.02$). In particular the lateral and vault location were more frequent in GPG than in BPG.

6. *Regional lymph node involvement* was more frequent in BPG than GPG ($P<0.01$).

7. *Metastasis* was less frequent in GPG than in BPG ($P<0.01$).

8. *Techniques of urinary diversion* were different in the two groups ($P<0.05$). GPG patients were more likely to have undergone a diversion through the ileal conduit and less likely to have had a ureterocutaneous diversion than BPG patients.

9. *Renal insufficiency* was less frequent in GPG than in BPG ($P<0.01$).

Four factors were found not to be prognostic *viz.*: sex, multiplicity of the tumour, histopathology of the tumour and presence of ova of schistosoma haematobium in the specimen (Table II).

### *Multivariate analysis*

In the first step of the multivariate analysis all the factors which proved to be of prognostic value from the univariate analysis were taken into account. For that purpose, the qualitative variables 'location of the tumour' and 'urinary diversion' were split into exclusive dichotomous variables (Table III). To further assess the relative prognostic value of each of the variables, successive discriminant analysis was performed in order to highlight the best discrimination, taking the Mahalanobis distance as a criterion.

The stepwise approach highlighted five preeminent prognostic factors, ranked as follows: grade, stage, renal insufficiency, diameter of the tumour and lymph node involvement (as in Table IV). The remaining four factors (age, metastasis, location of the tumour and urinary diversion) did not significantly increase the power of the discriminating function.

**Table III** Overall discriminant analysis between the two prognostic groups

| *Variable* | *Coding* | *Coefficient b* | *t* | *P* |
|---|---|---|---|---|
| Age (years) | continuous | 0.03 | 2.7 | 0.01 |
| Stage | 1 to 4 | 1.14 | 5.6 | 0.001 |
| Grade | 1 to 3 | 2.40 | 10.7 | 0.001 |
| Diameter of the tumour (cm) | continuous | 0.25 | 4.1 | 0.001 |
| Location of the tumour: | | | | |
| vault (Y/N) | 1/0 | −1.18 | 0.7 | NS |
| anterior (Y/N) | 1/0 | −0.06 | 2.5 | 0.02 |
| posterior (Y/N) | 1/0 | −0.90 | 0.2 | NS |
| lateral (Y/N) | 1/0 | −1.22 | 2.5 | 0.02 |
| Lymph node involvement (Y/N) | 1/0 | 0.69 | 3.1 | 0.01 |
| Metastasis (Y/N) | 1/0 | 1.48 | 2.8 | 0.01 |
| Renal insufficiency (Y/N) | 1/0 | 1.47 | 5.2 | 0.001 |
| Urinary diversion: | | | | |
| ileal conduit (Y/N) | 1/0 | −0.21 | 1.3 | NS |
| ileocecal bladder (Y/N) | 1/0 | −0.48 | 0.1 | NS |
| ureterocutaneous (Y/N) | 1/0 | 1.06 | 2.9 | 0.01 |

Discriminant analysis obtained with the 9 prognostic factors (see **Table I**). For dichotomous variables (Y/N) YES is coded as 1 and NO as 0. Other qualitative variables are split into exclusive dichotomous variables. For example, subjects with trigone location are those who are coded NO (0) for the four other locations, or subjects with rectal bladder as urinary diversion are those who are coded NO (0) for the three other types of diversion. The column *b* refers to the coefficients of the discriminant function. The column *t* gives the value of the test of comparison of the coefficient *b* to zero. The column *P* gives the level of significance by *t*-test.

**Table IV** Stepwise discriminant analysis

| *Variable* | *Coding* | *Coefficient b* | *t* | *P* |
|---|---|---|---|---|
| Grade | 1 to 3 | 2.36 | 10.7 | <0.001 |
| Stage | 1 to 4 | 1.31 | 5.6 | <0.001 |
| Renal insufficiency (Y/N) | 1/0 | 1.48 | 5.5 | <0.001 |
| Diameter of the tumour (cm) | continuous | 0.24 | 3.2 | <0.01 |
| Lymph node involvement (Y/N) | 1/0 | 0.96 | 2.7 | <0.01 |

This table shows the last step of the stepwise discriminant analysis. The five variables are shown in descending order of discriminating power.

The results of the final discriminant analysis with this last set of five factors are shown in Table IV.

A linear simplified discriminating score was determined as:

$X$ = 10 grade (1 to 3) + 5 stage (1 to 4) + 6 renal insufficiency (Y/N) + 1 diameter of the tumour (cm) + 4 lymph node involvement (Y/N)

The larger the score $X$, the greater was the probability of a bad prognosis for the patient.

Among all possible cut-off points, we have evaluated the midpoint (39) between the good prognosis mean score (33) and the bad prognosis mean score (44).

Therefore, assuming that patients with a score $\leq 39$ are classified as the good prognosis group and $>39$ as the bad prognosis group, we obtained the contingency table 'actual *versus* predicted status' (Table V). From this table it may be seen that $(126+120)/310=79.4\%$ would be correctly classified according to our model and assumption.

**Table V** Classification according to the score

| | *GPG* $\leq 39$ | *BPG* $>39$ | *Total* |
|---|---|---|---|
| GPG | 126 | 29 | 155 |
| BPG | 35 | 120 | 155 |
| Total | 161 | 149 | 310 |

### Discussion

The main aim of the present study was the evaluation of the prognostic value of 13 variables measured at the time of radical cystectomy of bilharzial bladder cancer subjects. Most prognostic studies of bladder cancer have dealt with survival (Osborn *et al.*, 1982; England *et al.*, 1981; Kishi *et al.*, 1981; Cifuentes Delatte *et al.*, 1982) and a minority with recurrence or metastasis (Dalesio *et al.*, 1983; Pocock *et al.*, 1982). Moreover these prognostic studies have generally been concerned with 'Western' bladder cancer (Osborn *et al.*, 1982; Dalesio *et al.*, 1983; Pocock *et al.*, 1982) while few studies have dealt with bilharzial bladder cancer (Ghoneim *et al.*, 1972, 1976, 1979; Sherif and Ibrahim, 1983).

Nine factors proved prognostic for recurrence in our study *viz*: age, pathological stage of the tumour, its size, location in the bladder, grade as well as regional lymph node involvement, distant metastasis in other organs, renal insufficiency and urinary diversion. Most of them also proved to be prognostic for survival (Osborne *et al* 1982; Cifuentes Delatte *et al* 1982; Dalesio *et al* 1983; Kishi *et al.*, 1981; England *et al.*, 1981; Ghoneim *et al.*, 1972, 1976; Smith & Whitmore, 1981; Varkarakis *et al.*, 1975; Ballanger & Ballanger, 1982). Our study did not prove the prognostic value of multiplicity or histopathology of the tumour; some authors concur with this conclusion while others dissent (Ghoneim *et al.*, 1979; Kishi *et al.*, 1981). Presence of ova of schistosoma haematobium in the tumour did not appear to be prognostic and this confirms the previous work of the present authors (Sherif & Ibrahim, 1983). Finally, contrary to the study of Osborn *et al.* (1982), we did not find a correlation between sex and the prognosis of bilharzial bladder cancer.

Linear discriminant analysis has been used in this study to take into account the relationship between the prognostic variables. We have checked by other techniques that our results would not be significantly altered of we chose a dichotomous coding to code the semi-quantitative variables as grade and stage.

In conclusion, we have determined two extreme profiles for bilharzial bladder cancer subjects: Good prognosis patients are those with a superficial tumour of the well differentiated type with tumour <5 cm in diameter, with normal renal function and without lymph node involvement. Patients wirh poorly differentiated infiltrating tumour, >5 cm in diameter, with renal insufficiency and with lymph node involvement have the worst prognosis.

Prediction of outcome by utilizing our model is a point of considerable clinical importance. Assignment to the bad prognosis group should alert treating surgeons to the need for more frequent follow-up of their patients and for continuity of management.

### References

BALLANGER, R. & BALLANGER, PH. (1982). Insuffisance renale chronique par cancer vesical. *Ann. Urol.*, **16**, 165.

CIFUENTES DELATTE, L., GARCIA DE LA PENA, E. & VELA NAVARRETE, R. (1982). Survival rates of patients with bladder tumours. An Experience of 1,744 cases (1950–1978). *Br. J. Urol.*, **54**, 267.

DALSIO, O., SCHULMAN, C.C., SYLVESTER, R. & 6 others (1983). Prognostic factors in superficial bladder tumors. A study of the European organization for research and treatment of cancer: Genitourinary Tract Cancer Cooperative Group. *J. Urol.*, **129**, 730.

EL-BOLKAINY, M. & CHU, E. (1981). In *Detection of bladder cancer associated with schistosomiasis,* El-Bolkainy, M. & Chu, E. (eds) p. 122. Al-Ahram Press: Cairo.

EL-SEBAI, I. (1981). Carcinoma of the urinary bladder in Egypt, current clinical experience. In *Detection of bladder cancer associated with schistosomiasis,* El-Bolkainy, M. & Chu, E. (eds) p. 9. Al-Ahram Press: Cairo.

EL-SEBAI, I. (1983). Clinical aspects, staging and management of cancer of the bilharzial bladder. In *Bladder cancer,* Vol. **II,** Cancer of the bilharzial bladder, El-Sebai (ed) p. 29. CRC Press: Florida.

ENGLAND, H.R., PARIS, A.M.I. & BLANDY, J.P. (1981). The correlation of T1 bladder tumour history with prognosis and follow-up requirements. *Br. J. Urol.,* **53,** 593.

FLEISS, J.L. (1981). *Statistical Methods for Rates and Proportions,* John Wiley & Sons: New York.

GHONEIM, M.A., MANSOUR, M.A. & EL-BOLKAINY, M.N. (1972). Radical cystectomy for carcinoma of the bilharzial bladder. *Br. J. Urol.,* **44,** 461.

GHONEIM, M.A., EL-BOLKAINY, M.N., MANSOUR, M.A., EL-HAMMADY, S.M. & ASHAMALLAH, A.G. (1976). Radical cystectomy for carcinoma of the bilharzial bladder: Technique and results. *Urology,* **8,** 547.

GHONEIM, M.A., ASHAMALLAH, A.G., EL-HAMMADY, S., GABALLAH, M.A. & SOLIMAN, E.S. (1979). Cystectomy for carcinoma of the bilharzial bladder: 138 cases 5 years later. *Br. J. Urol.,* **51,** 541.

IBRAHIM, A.S. (1981). Epidemiology of cancer of the urinary bladder in Egypt. Proc. first UICC conference on cancer prevention in developing countries. Nayoga, Japan (abstract).

KISHI, K., HIROTA, T., MATSUMOTO, K., KAKIZOE, T., MURASE, T. & FUJITA, J. (1981). Carcinoma of the bladder: A clinical and pathological analysis of 87 autopsy cases. *J. Urol.,* **125,** 36.

LACHENBRUCH, PETER A. (1975). *Discriminant analysis,* Hafner Press: New York.

OSBORN, D.E., HONAN, R.P., PALMER, M.K., BARNARD, R.J., McINTYRE, D. & POINTON, R.S. (1982). Factors influencing salvage cystectomy results. *Br. J. Urol.,* **54,** 122.

POCOCK, R.D., PONDER, B.A.J., O'SULLIVAN, J.P., IBRAHIM, S.K., EASTON, D.F. & SHEARER, R.J. (1982). Prognostic factors in non-infiltrating carcinoma of the bladder: A preliminary report. *Br. J. Urol.,* **54,** 711.

SCHWARTZ, D. (1980). *Methodes statistiques a l'usage des medecins et des biologistes.* Edition Flammarion: Paris, France.

SHERIF, M. & IBRAHIM, A.S. (1983). Prognostic factors in bilharzial bladder cancer. Proc. WHO conference on urinary bladder cancer. Edsmyr, F. & Anderson, L. (eds) Montedison Lakemedel AB: Stockholm, Sweden.

SMITH, J.A. & WHITMORE, W.F. (1981). Regional lymph node metastasis from bladder cancer *J. Urol.,* **126,** 591.

UICC. UNION INTERNATIONALE CONTRE LE CANCER (1979). *TNM Classification des tumeurs malignes,* Third edition. International Union against Cancer: Geneva.

VARKARAKIS, M.J., GAETA, J., MOORE, R.H. & MURPHY, G.P. (1975). Prognosis of bladder carcinoma in patients treated with cystectomy. *Int. Urol. Nephrol,* **7,** 39.